# Comparative evaluation of lightweight and pre-trained deep learning models for multi-class classification of infected freshwater fish species in Thailand

**DOI:** 10.14202/vetworld.2026.1215-1228

**Published:** 2026-03-23

**Authors:** Sivaramasamy Elayaraja, Satish Nandipati, Vlastimil Stejskal, Channarong Rodkhum

**Affiliations:** 1 Center of Excellence in Fish Infectious Diseases (CE FID), Faculty of Veterinary Science, Chulalongkorn University, Bangkok 10330, Thailand; 2 University of South Bohemia in České Budějovice, Faculty of Fisheries and Protection of Waters, South Bohemian Research Center of Aquaculture and Biodiversity of Hydrocenoses, Institute of Aquaculture and Protection of Waters, České Budějovice 370 05, Czech Republic; 3 Faculty of Sciences, University of South Bohemia, České Budějovice (Budweis), Czech Republic; 4 Embrear Scientific, Houston, Texas 77077, USA

**Keywords:** aquaculture disease detection, convolutional neural network, deep learning, fish disease classification, image-based diagnosis, infected freshwater fish, InceptionV3, machine learning in aquaculture

## Abstract

**Background and Aim:**

Aquaculture plays a crucial role in global food security; however, disease outbreaks remain a major constraint to sustainable production. Rapid and reliable detection of fish diseases is essential to reduce mortality, economic losses, and the misuse of antimicrobials in aquaculture systems. Conventional diagnostic approaches, such as clinical observation and bacterial culturing, are time-consuming, costly, and require specialized expertise. Recent advances in deep learning, particularly convolutional neural networks (CNNs), have shown promise in automating image-based disease detection. This study aimed to compare a lightweight three-layer CNN model with pre-trained deep learning architectures (VGG16, InceptionV3, and ResNet50) for multi-class classification of infected freshwater fish species using a balanced image dataset collected from aquaculture farms in Thailand.

**Materials and Methods:**

Images from clinically infected freshwater fish were collected during routine farm inspections across six provinces in Thailand. The dataset included 424 images of four species: Asian seabass (*Lates calcarifer*), red tilapia (*Oreochromis* sp.), snakeskin gourami (*Trichopodus pectoralis*), and snakeheads (*Channa striata*). After preprocessing, a balanced dataset of 56 images per class (totaling 224) was created. The dataset was divided into training (80%) and testing (20%) subsets. On-the-fly data augmentation techniques, such as rotation, brightness adjustment, flipping, shifting, shearing, and zooming, were applied to the training data to reduce overfitting. A lightweight, three-layer CNN model with stochastic gradient descent was used and compared with pre-trained architectures (VGG16, InceptionV3, and ResNet50). Model performance was assessed through accuracy, precision, recall, F1-score, confusion matrix analysis, and five-fold cross-validation.

**Results:**

Among the evaluated models, InceptionV3 achieved the highest classification accuracy (56.82%), followed by VGG16 (43.18%) and the proposed CNN (38.64%), while ResNet50 performed poorly (25%). The InceptionV3 model also demonstrated higher average precision (63%), recall (57%), and F1-score (56.75%), indicating superior classification capabilities. Confusion matrix analysis revealed that InceptionV3 correctly classified 25 out of 44 test images, outperforming the proposed CNN (17 correct predictions) and VGG16 (19 correct predictions). Five-fold cross-validation further confirmed the stability and relatively better performance of the InceptionV3 model.

**Conclusion:**

The comparative evaluation shows that pre-trained CNN architectures, especially InceptionV3, outperform a lightweight three-layer CNN when trained on small, balanced datasets of infected fish images. Although the proposed lightweight CNN has limited accuracy, its low computational needs suggest it could be useful in resource-limited aquaculture settings. Incorporating deep learning–based image analysis into aquaculture health monitoring systems could enable quick disease triage, support timely management decisions, and promote better biosecurity and sustainable fish production. Future research should increase the dataset size, include more fish species and disease types, and test model performance across different farming environments to improve its generalizability and practical use.

## INTRODUCTION

Aquaculture is a vital food production sector that drives economic growth and global food security. The sharp rise in seafood consumption has speed up aquaculture development, prompting producers to increase stock densities and adopt operational efficiencies to satisfy market demands [1]. This rapid growth has boosted the production of key aquaculture species such as seabass, tilapia, snake gourami, and salmon. According to recent data from the Food and Agriculture Organization (FAO) (2024), aquaculture now makes up more than half of all fish consumed worldwide. In 2022, aquaculture production hit a record 130.9 million tonnes, surpassing capture fisheries for the first time and accounting for over half of all aquatic animal production [2, 3].

While this expansion offers significant opportunities, it also heightens the need for effective disease prevention and health management. Intensive farming systems are especially vulnerable to disease outbreaks due to high stocking densities and shared water environments [4, 5]. Among the various health challenges in aquaculture, bacterial infections are some of the most common diseases affecting aquatic species. Several bacterial pathogens frequently linked to disease outbreaks in fish include *Aeromonas* (furunculosis), *Vibrio* (vibriosis), *Streptococcus* (streptococcosis), *Staphylococcus*, *Edwardsiella* (edwardsiellosis), Flavobacterium (columnaris disease), *Francisella* sp. (francisellosis), *Yersinia* sp. (enteric redmouth disease), and *Renibacterium* (bacterial kidney disease) [6].

These infections can lead to significant economic losses due to high mortality rates, reduced growth performance, and lower fish quality. Clinical signs often include skin lesions, hemorrhages, fin rot, or damage to internal organs, ultimately decreasing fish health and productivity. The economic impact of bacterial infections is therefore substantial, affecting both small-scale and commercial aquaculture operations. Additionally, disease outbreaks involving zoonotic bacteria can disrupt trade and pose public health risks [7]. Environmental stressors such as poor water quality, temperature changes, and overcrowding further worsen disease outbreaks, emphasizing the need for effective health management strategies to support sustainable aquaculture production [8].

Early and precise diagnosis of fish infections is essential for preventing outbreaks and reducing economic losses. Traditional diagnostic methods, such as clinical observation and bacterial culturing, are common but often slow and may lack diagnostic accuracy [9]. Molecular and immunological diagnostic tools, like polymerase chain reaction (PCR) and enzyme-linked immunosorbent assay, have enhanced the speed and precision of diagnosis. Furthermore, next-generation sequencing technologies now enable advanced pathogen identification and antimicrobial resistance monitoring [10–12]. Despite these advancements, many aquaculture farms still encounter practical challenges, including high costs, lengthy diagnostic turnaround times, and the requirement for specialized laboratories and trained staff. As a result, there is an increasing need for rapid, affordable, and field-ready screening techniques that can facilitate early disease detection and support confirmatory laboratory testing.

Convolutional neural networks (CNNs), a class of deep learning (DL) algorithms, have recently shown remarkable success in computer vision and image processing applications. These models can automatically extract complex features from images, enabling accurate classification, object recognition, and semantic segmentation while reducing the need for manual feature engineering [13, 14]. Several studies have examined the use of CNNs for fish species identification and disease detection. For example, the IsVoNet8 CNN model has been used to classify eight marine fish species across six families, achieving an accuracy of 98.62%, outperforming models such as ResNet50 (91.37%), ResNet101 (86.12%), and VGG16 (97.75%) [15]. In another study, DL approaches were applied to detect wounds and lice in Atlantic salmon, where a 15-layer CNN model achieved an accuracy of 96.7%, surpassing VGG-19 (91.2%) and VGG-16 (92.8%) models [16]. Similarly, machine learning methods have been employed to classify fish diseases such as epizootic ulcerative syndrome, fin rot, and tail rot, with the random forest algorithm reaching an accuracy of 88.87% and outperforming other tested models [17].

Despite rapid advancements in artificial intelligence–based tools for aquaculture monitoring, several limitations still exist in the current research. Most existing studies on fish disease detection using DL depend on large, curated datasets with thousands of images collected under controlled conditions. These datasets often feature clearly separated disease categories or focus on binary classification tasks like healthy versus diseased fish. In contrast, real-world aquaculture environments usually produce smaller, diverse image datasets that vary in lighting, background, camera angle, and lesion presentation. Additionally, many previous studies have mainly concentrated on marine species or publicly available datasets rather than images collected directly from commercial aquaculture farms. Consequently, the application of these models to real farm conditions remains uncertain.

Another limitation is that many studies focus on high-performing, computationally demanding DL architectures without considering their feasibility in low-resource settings. Many aquaculture farms, especially in developing regions, lack access to high-performance computing infrastructure, which limits the practical use of complex DL models. Lightweight CNN architectures that need fewer computational resources have been suggested as alternatives; however, their comparative performance against established pre-trained models is not well understood. Additionally, previous research has seldom evaluated balanced multi-class datasets of infected freshwater fish species that show common bacterial disease signs observed during regular farm inspections. The scarcity of such datasets, particularly from Southeast Asian aquaculture systems, highlights a significant knowledge gap in applying DL for fish health monitoring.

Therefore, this study aimed to compare the performance of a lightweight three-layer CNN with several popular pre-trained DL architectures, including VGG16, InceptionV3, and ResNet50, for multi-class classification of infected freshwater fish species from Thailand. Specifically, the study used a balanced image dataset representing four commonly cultured freshwater fish species, Asian seabass (*Lates calcarifer*), red tilapia (*Oreochromis* sp.), snakeskin gourami (*Trichopodus pectoralis*), and snakeheads (*Channa striata*), collected from commercial aquaculture farms across multiple provinces in Thailand. The research also aimed to determine if a lightweight CNN model could serve as a practical solution for disease classification in resource-limited aquaculture settings while still providing acceptable predictive accuracy. By comparing lightweight and pre-trained architectures with standardized evaluation metrics, this study seeks to offer insights into the suitability of various DL approaches for practical aquaculture disease monitoring and to support the development of quick, image-based diagnostic tools that promote sustainable aquaculture health management.

## MATERIALS AND METHODS

### Ethical approval

All procedures performed in this study were conducted in accordance with internationally accepted ethical standards for the care and use of animals in scientific research. The study protocol, including field sampling, fish handling, image acquisition, and laboratory diagnostic procedures, was reviewed and approved by the Animal Care and Use Committee for Scientific Research at Kasetsart University, Thailand (approval numbers: ACKU64-FI8-007, ACKU64/F1S/008, ACKU64-FI8-009, and ACKU-FIS-055). In addition, the biosafety protocol and laboratory procedures were implemented in accordance with the regulations of the Faculty of Veterinary Science Committee, Chulalongkorn University, Bangkok, Thailand.

All images used in this study were obtained during routine health monitoring visits to commercial aquaculture farms located in Kanchanaburi, Samut Sakhon, Suphanburi, Ratchaburi, Phetchaburi, and Nakhon Pathom provinces, Thailand. Written permission was obtained from farm owners prior to sample observation and photography. No fish were intentionally euthanized or experimentally infected for the purposes of this research. The images were captured during standard farm operations while fish were being handled by farm personnel as part of routine health inspections.

Handling of fish during image acquisition followed recommended aquaculture welfare guidelines to minimize stress and injury. Fish were briefly restrained only when necessary for documentation of external clinical signs and were returned immediately to their respective culture systems after image capture. All investigators adhered to farm-level biosecurity procedures, including the use of protective clothing, hand sanitation, and equipment disinfection to prevent cross-contamination between culture units.

Where laboratory confirmation was performed, diagnostic procedures such as microbiological culture, Gram staining, matrix-assisted laser desorption/ionization time-of-flight mass spectrometry (MALDI-TOF MS), and PCR were conducted following standard microbiological and molecular diagnostic protocols. All laboratory work was performed under appropriate biosafety conditions in accordance with institutional biosafety regulations.

Because the study relied exclusively on observational image data collected during routine farm health assessments and did not involve experimental manipulation, infection challenge, or euthanasia of animals, the procedures were considered minimal risk and compliant with institutional animal welfare policies.

### Study period and location

This study was conducted from March 2021 to April 2024 at the Center of Excellence in Fish Infectious Diseases (CE-FID) Laboratory, Faculty of Veterinary Science, Chulalongkorn University, Bangkok, Thailand, using samples and images collected through field sampling at freshwater aquaculture farms across multiple provinces in Thailand.

### Data source, diagnostic confirmation, and inclusion and exclusion criteria

Following ethical approval and permission, samples were collected during routine health assessments from commercial freshwater farms located in Kanchanaburi, Samut Sakhon, Suphanburi, Ratchaburi, Phetchaburi, and Nakhon Pathom, Thailand. During farm visits, *Aeromonas*, *Vibrio*, *Streptococcus*, and *Flavobacterium* were commonly detected at various diagnostic levels. However, viral and parasitic presentations were rare and were not systematically confirmed. Therefore, the dataset in this study primarily focuses on bacterial-type lesions with lot-level laboratory support where available.

Fish exhibiting one or more common clinical signs, including skin ulcers, hemorrhagic spots, fin rot, scale loss, or exophthalmia, were classified as infected fish (i.e., clinically infected fish). This classification was determined through an initial clinical screening independently performed by two aquatic health specialists. Laboratory-supported fish were defined as those in which at least one laboratory technique, such as microbiological plating, Gram staining, matrix-assisted laser desorption/ionization time-of-flight mass spectrometry (MALDI-TOF MS) for colony identification, or PCR, was employed when necessary to confirm the clinical findings.

The exclusion criteria included cases where species identification was not possible, essential anatomical features were obscured, images were extremely blurry, or images were over- or underexposed regarding brightness or contrast.

Instead of attributing infections to individual fish, the variability in multi-pathogen lesions shown in these images offers realistic visual diversity that enhances the dataset’s uniqueness and improves model robustness. As a result, the DL input dataset consisted of fish images with multi-pathogen lesions, collected from both clinical and experimental environments.

### Image acquisition and preprocessing of data

**Figure 1:**
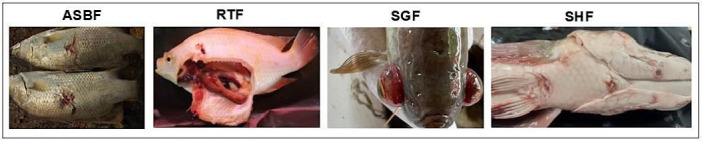
Representative digital images of infected freshwater fish species used in this study, including Asian seabass (ASBF), red tilapia (RTF), snakeskin gourami (SGF), and snakeheads (SHF).

Images were captured using a Nikon C5300 camera (Nikon Corporation, Tokyo, Japan) under natural daylight or barn lighting conditions, primarily from a lateral view. When handling conditions permitted, additional viewpoints were also collected; however, the standard imaging orientation remained lateral. Image acquisition was conducted during routine farm activities following established biosecurity protocols to minimize fish stress (Figure 1).

The dataset of infected freshwater fish species included four multi-class categories with a total of 424 bacterial-infected fish images. These categories were Asian seabass (*Lates calcarifer*; abbreviated as ASBF; 125 images), red tilapia (*Oreochromis* sp.; RTF; 180 images), snakeskin gourami (*Trichopodus pectoralis*; SGF; 56 images), and snakeheads (*Channa striata*; SHF; 63 images). The images ranged from 252 to 281 pixels in width and 4608 pixels in height (Figure 1).

Several preprocessing steps were taken to enhance image quality. These included manually cropping to remove unwanted areas around the image and labels. Background removal was not done. Since DL models often overfit when trained with small datasets, this poses a challenge for DL applications. One way to address this is to artificially increase the dataset size. Consequently, a larger datasets can boost DL performance, and image augmentation is one technique for this.

In this study, on-the-fly image augmentation was applied to the training dataset. The augmentation techniques included the following:

Brightness adjustment: Images with varying brightness levels were generated.

Flip: This data augmentation technique involves flipping the matrix’s columns and rows either horizontally or vertically. Exposing the model to different orientations of the same image can improve classification accuracy.

Rotation: Rotation is a commonly used augmentation technique where the original image is randomly rotated clockwise or counterclockwise by a set number of degrees to change its position within the frame.

Shearing: Shear transformation, also known as image slanting, involves stretching one axis at a specific angle. The shearing angle is given in degrees as a floating-point number.

Shift: Images can be moved up, down, left, or right by a set number of degrees to change their position within the frame.

Zoom: Zoom augmentation produces images at various zoom levels to increase dataset diversity.

### Training and testing datasets

**Figure 2:**
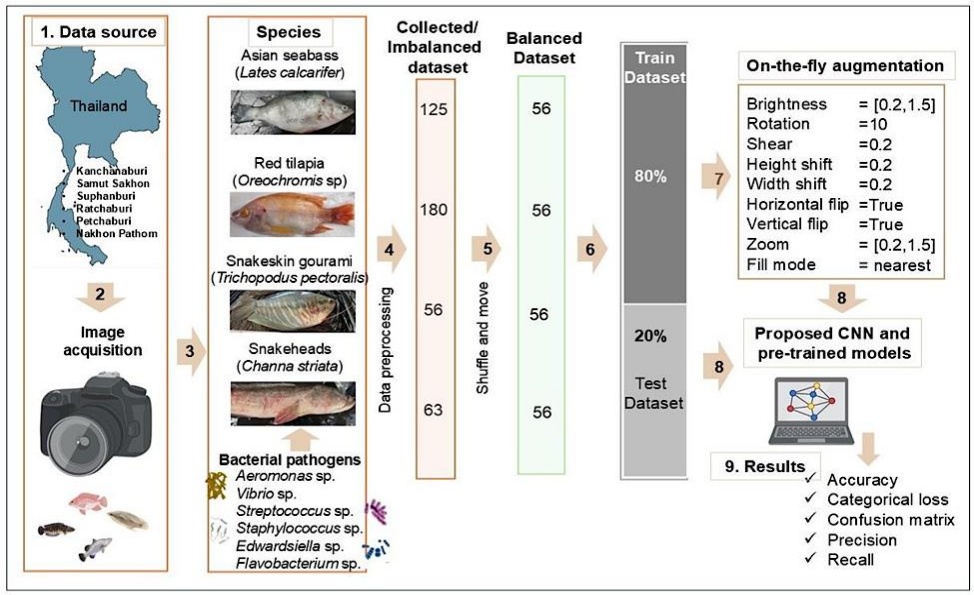
Workflow of the deep learning classification pipeline. The workflow includes: (1) data collection from aquaculture farms across different provinces in Thailand; (2) image acquisition using a Nikon C5300 camera; (3) selection of multi-class freshwater fish species; (4) image preprocessing, including cropping; (5) dataset balancing using bash “shuffle and move” commands; (6) dataset split into training and testing sets (80–20%); (7) application of on-the-fly data augmentation to the training dataset; (8) model input to the proposed convolutional neural networks and pre-trained models (VGG16, InceptionV3, and ResNet50); and (9) evaluation using multi-class statistical metrics including accuracy, categorical loss, confusion matrix, precision, recall, and F1-score.

Figure 2 shows the uneven distribution of infected freshwater fish images across the four classes, indicating an imbalanced dataset. In such situations, the classifier usually favors the class with the most instances. Therefore, it is important to pay special attention to datasets that have class imbalance [18].

Although ensemble classifiers can enhance classification performance, they are not effectively applicable to imbalanced datasets due to their static nature [19]. Additionally, experimental results have shown that balanced datasets perform better than imbalanced ones [20, 21].

In the current study, the imbalanced dataset of preprocessed images was converted into a balanced one. The snakeskin gourami (SGF), which had the fewest images (56), served as the baseline for balancing all classes. As a result, 56 images were selected for each class, creating a balanced dataset of 224 images across four classes.

The dataset was divided into training and testing subsets with an 80%–20% split. The training set included 45 images per class, while the testing set had 11 images per class. The dataset balancing was done using the “shuffle and move” bash script to reduce the chance of data leakage.

On-the-fly augmentation was applied to the training dataset with the following parameters: rotation (10°), width shift (0.2), height shift (0.2), horizontal and vertical flips (True), brightness range [0.2–1.5], shear (0.2), zoom range [0.2–1.5], fill mode (nearest), and seed value set to 42 (Figure 2).

### Proposed lightweight three-layer CNN

In this study, CNNs were used for multi-class classification of infected freshwater fish species. The models were implemented using Python (v. 3.12.12, https://www.python.org/downloads/) and the Keras API (v. 3.10.0, https://pypi.org/project/keras/), running on the TensorFlow framework (v. 2.19.0, https://www.tensorflow.org/).

**Figure 3:**
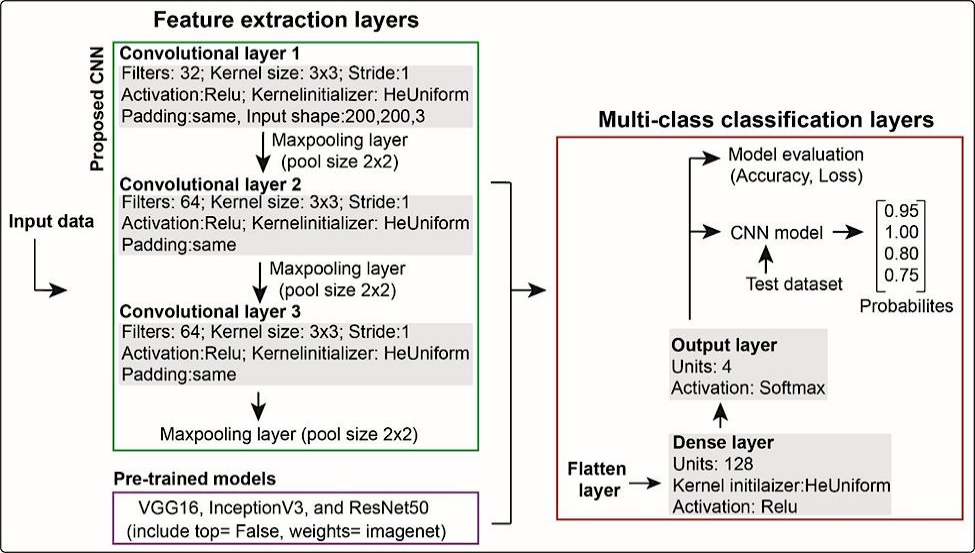
Architecture of the convolutional neural network (CNN) and pre-trained models used in this study. The proposed CNN has three convolutional layers with max-pooling for feature extraction, followed by classification layers including flatten, dense, and output layers. Pre-trained models (with top = False and weights = ImageNet) were fine-tuned using the same classification layers for multi-class prediction.

Figure 3 shows the architecture pipeline that includes input images, feature extraction layers, and classifier layers. The proposed lightweight CNN architecture has three convolutional layers. The first convolutional layer uses 32 filters with an input shape of 200 × 200 × 3 (used for both the CNN and pre-trained models). The second and third convolutional layers have 64 and 128 filters, respectively.

Additional parameters included padding set to “same,” activation function using the rectified linear unit (ReLU), kernel size of 3 × 3, stride of one pixel, and kernel initializer set to HeUniform. These convolutional layers were followed by default max-pooling layers with a pool size of 2 × 2 for feature extraction.

The classification component included a flattened layer, a dense layer with 128 units using ReLU activation, and a four-class output layer with softmax activation for classification. This configuration was used for both the proposed CNN and the pre-trained models.

The stochastic gradient descent (SGD) optimizer was employed with a learning rate of 0.001 and a momentum of 0.9. Preliminary analyses (results not shown) indicated that SGD outperformed the Adam optimizer in this study. The model was compiled using categorical cross-entropy as the loss function and categorical accuracy as the evaluation metric, with training conducted for 20 epochs and a batch size of 64. Poor performance was observed with a batch size of 32 and either 15 or 20 epochs.

The proposed CNN model was compared to three pre-trained architectures: VGG16, ResNet50, and InceptionV3. For these models, the parameters included top = False (indicating the classification layers of the original models were not loaded) and weights = ImageNet, while the top layers were kept for fine-tuning.

The input dataset for both CNN and pre-trained models included 224 images across four classes, split 80% for training and 20% for testing. Since image classification requires significant computational resources, model training was conducted using Google Colab with GPU access (Tesla T4, 15 GB RAM) and 112.6 GB of disk space. The system configuration featured NVIDIA-SMI 550.54.15, driver version 550.54.15, and CUDA version 12.4, with all necessary packages installed [22].

### Evaluation of data and GPU performance

The evaluation of the proposed CNN and the pre-trained models used a training-testing approach. A total of 224 images across four classes were split into two groups: 80% for training (180 images; 45 per class) and 20% for testing (44 images; 11 per class).

Model performance on the test dataset was evaluated using multi-class statistical metrics, including accuracy, precision, recall, and F1-score (Figure 2). GPU performance parameters such as temperature, power consumption, memory usage, and GPU utilization were tracked using the NVIDIA System Management Interface (NVIDIA-SMI).

Accuracy measures the proportion of correctly classified samples, including true positives (TP) and true negatives (TN), out of all samples in the dataset (which also includes false positives [FP] and false negatives [FN]).

Accuracy = (TP + TN) / (TP + FP + TN + FN)

Categorical cross-entropy loss: This metric evaluates the difference between predicted and actual probability distributions [23].

CE Loss = −1/N Σ_i=1_^N^ Σ_j=1_^c^ Y_ij_ log(p_ij_)

Precision: Precision measures the proportion of true positive predictions (TP) out of all predicted positives (TP + FP).

Precision = TP / (TP + FP) × 100%

Recall: Recall indicates the proportion of correctly predicted positive samples compared to the total number of actual positive samples.

Recall = TP / (TP + FN) × 100%

F1 score: The F1 score is the harmonic mean of precision and recall, accounting for both FP and false negatives.

F1 score = 2 × (Precision × Recall) / (Precision + Recall)

Confusion matrix: The confusion matrix is a table used to visualize how well a classifier performs by comparing predicted labels with actual labels.

## RESULTS

### Model performance evaluation

**Figure 4:**
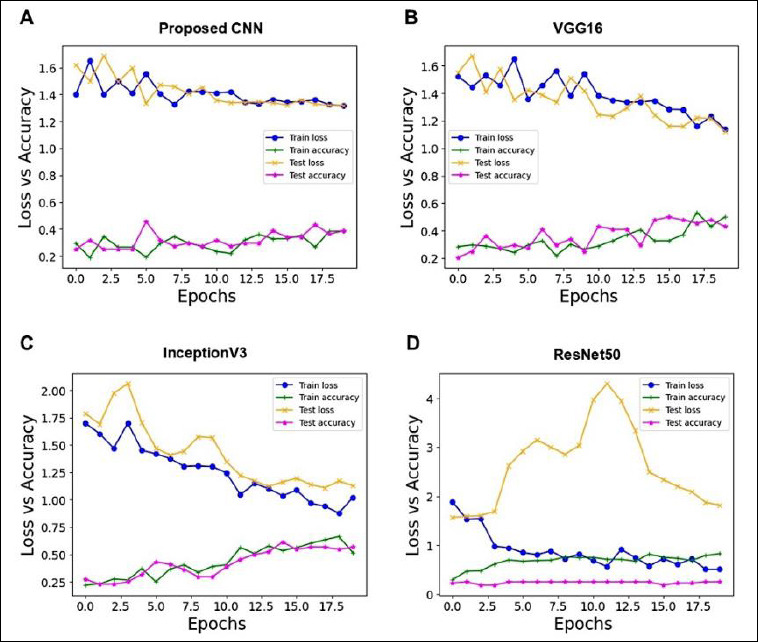
Training and testing loss and accuracy curves of each model over 20 epochs. (A) Proposed convolutional neural networks. (B) VGG16. (C) InceptionV3. (D) ResNet50. Train loss (blue solid line with dots) and test loss (orange solid line with × markers), train accuracy (green solid line with + markers), and test accuracy (pink solid line with * markers) are shown. The x-axis represents the number of training epochs, and the y-axis represents loss and accuracy values.

The accuracy and loss curves after 20 epochs of training and testing datasets were used to evaluate the performance of the proposed CNN model and the pre-trained models (Figures 4A–D). The test dataset accuracy was 38.64% for the proposed CNN, 43.18% for VGG16, and 56.82% for InceptionV3. Among all four models, ResNet50 performed poorly, with an accuracy of 25% compared to the proposed CNN and other pre-trained models; therefore, this model was excluded from further analysis.

The proposed CNN showed no consistent decrease in training and testing loss and exhibited inconsistent accuracy patterns, indicating that the model did not adequately fit the data (Figure 4A). However, minor fluctuations were observed in the loss and accuracy curves for both training and testing datasets. A steady decline in loss suggests that the model fit the data reasonably well, and because there were no signs of training accuracy increasing substantially beyond test accuracy, the dataset was neither overfitted nor underfitted. Under these conditions, the VGG16 and InceptionV3 models demonstrated improved learning performance (Figures 4B–C).

### Classification model performance

Table 1 shows the ranges and average values of precision, recall, and F1 score (classification report) for each model, along with overall accuracy and GPU performance metrics. The proposed CNN achieved an accuracy of 38.64%, with precision ranging from 0–100% (average: 42%), recall ranging from 0%–73% (average: 38.7%), and F1-score ranging from 0%–46% (average: 33%). The training runtime was 128.65 s, and the GPU performance metrics (temperature, power, memory usage, and GPU utilization) were 52°C, 30 W, 4474 MiB, and 0%, respectively.

**Table 1:** Classification performance metrics and GPU resource utilization for all evaluated models.

Metric	3CNN	VGG16	InceptionV3	ResNet50
Accuracy (%)	38.64	43.18	56.82	25
Precision (%)	0–100	33–70	42–100	0–25
Average precision	42	43.5	63	6.25
Recall (%)	0–73	9–64	27–73	0–100
Average recall	38.7	43.25	57	25
F1-score (%)	0–46	14–67	38–84	0–40
Avg F1-score	33	41	56.75	10
Run time (seconds)	128.65	295.37	253.17	122.08
Temperature (°C)	52	77	76	66
Power (W)	30	44	44	36
Memory usage (MiB)	4474	14064	8350	1409
GPU utilization (%)	0	72	23	0

The VGG16 model achieved an accuracy of 43.18%, with precision ranging from 33% to 70% (average: 43.5%), recall ranging from 9% to 64% (average: 43.25%), and F1-score ranging from 14% to 67% (average: 41%). The training runtime was 295.37 s. GPU performance metrics were 77°C temperature, 44 W power consumption, 14064 MiB memory usage, and 72% GPU utilization.

Similarly, InceptionV3 achieved the highest accuracy of 56.82%, with precision ranging from 42% to 100% (average: 63%), recall ranging from 27% to 73% (average: 57%), and F1-score ranging from 38% to 84% (average: 56.75%). GPU performance metrics included a temperature of 76°C, power consumption of 44 W, memory usage of 8350 MiB, and GPU utilization of 23%, with a training runtime of 122.08 s.

Overall, the averages of precision, recall, F1-score, accuracy values, and GPU performance metrics show that InceptionV3 and VGG16 had better classification performance compared to the proposed CNN model.

### Five-fold cross-validation of model performance

The performance evaluation of the proposed CNN across five-fold cross-validation demonstrated an overall accuracy of 38.64%, with accuracy values ranging from 25% to 36.11%. Precision ranged from 0% to 60%, recall from 0% to 100%, and F1-score from 0% to 60%.

The average GPU performance during five-fold cross-validation was 52.4°C temperature, 34.2 W power consumption, and 3450 MiB memory usage, with GPU utilization ranging from 0% to 7%. The average training runtime was approximately 262 s.

In contrast, InceptionV3, which performed best in this study with an overall accuracy of 56.82%, showed accuracy values ranging from 47.22% to 58.33%. Precision ranged from 0% to 100%, recall ranged from 0% to 100%, and F1-score ranged from 0% to 67%. The average GPU performance during five-fold cross-validation was 75°C, 38.6 W, and 8,354.4 MiB, with GPU utilization reaching 16% in one fold. The training runtime ranged from 248.22 s to 842.89 s (Table 2).

**Table 2:** Five-fold cross-validation results and GPU performance metrics for the proposed CNN and InceptionV3 models.

Metrics/GPU	CNN Fold 1	Fold 2	Fold 3	Fold 4	Fold 5	InceptionV3 Fold 1	Fold 2	Fold 3	Fold 4	Fold 5
Accuracy (%)	33.33	25	30.56	30.56	36.11	50	55.56	50	47.22	58.33
Precision (%)	0–60	0–33	0–45	0–38	0–60	38–100	29–70	36–100	0–100	33–100
Recall (%)	0–100	0–86	0–86	0–83	0–91	43–100	50–71	17–67	0–56	44–100
F1-score (%)	0–45	0–41	0–45	0–43	0–60	27–67	36–62	25–57	0–53	52–67
Support	9	7–10	7–11	6–11	5–11	6–12	4–13	6–12	5–12	5–11
Run time (seconds)	149.65	205.97	262.31	318.03	374.37	248.22	391.45	539.74	679.08	842.89
Temperature (°C)	52	52	52	53	53	75	75	75	75	74
Power (W)	42	28	28	32	41	45	35	37	37	39
Memory usage (MiB)	3450	3450	3450	3450	3452	8348	8350	8354	8358	8362
GPU utilization (%)	0	0	7	0	3	16	0	0	0	0

### Evaluation of model performance on test data

The prediction on the four-class test dataset included 44 images, with 11 images in each class. The proposed CNN, with an accuracy of 38.64% and a recall range of 0%–73% (Table 1), correctly predicted 17 of 44 images (38.6%) across all classes except SHF.

**Figure 5:**
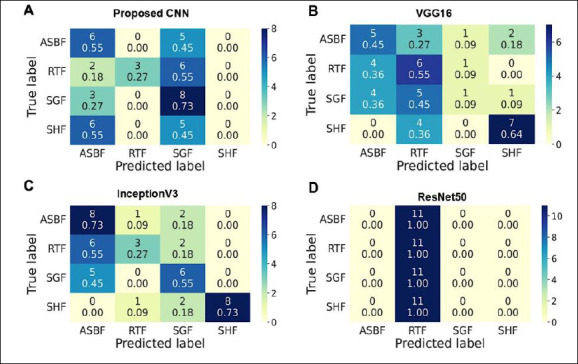
Confusion matrices showing the model’s classification performance. Rows indicate the actual class labels, while columns show the predicted class labels. A color bar (0–10 scale) represents the number of predictions. Diagonal cells signify correct predictions (true positives and true negatives), whereas off-diagonal cells indicate misclassifications (false positives and false negatives). Each cell displays the raw image count at the top and the normalized value (recall proportion) at the bottom.

Among the 11 images per class, correctly predicted images included ASBF (6 images, 55%), RTF (3 images, 27%), and SHF (8 images, 73%). The ASBF, RTF, and SGF classes were often misclassified as other classes, with misclassification ranging from 2 to 6 images (18%–55%). Meanwhile, SHF images were not consistently classified correctly, indicating overall poor model performance (Figure 5A).

The VGG16 model, with a recall range of 9%–64% and an accuracy of 43.18% (Table 1), correctly predicted 19 out of 44 images (43.18%). Correct predictions included ASBF (5 images, 45%), RTF (6 images, 55%), SHF (1 image, 9%), and SGF (7 images, 64%). Misclassification across classes involved 1 to 5 images (9%–45%) (Figure 5B).

In contrast, InceptionV3 correctly predicted 25 out of 44 images (Figure 5C), resulting in an accuracy of 56.82%, with a recall range of 27%–73% (Table 1). Correct predictions were made for ASBF and SHF (8 images each, 73%), RTF (3 images, 27%), and SGF (6 images, 55%). Misclassification across classes ranged from 3 to 8 images (9%–55%).

Overall, the results show that the InceptionV3 and VGG16 models, along with the proposed CNN model (except for the SHF class), accurately classified the test dataset into multiple categories. These findings indicate that these models could be useful for classifying infected freshwater fish species.

## DISCUSSION

### Importance of disease detection in aquaculture

In aquaculture, maintaining fish health, ensuring high production yields, and reducing financial losses depend on effective disease management. In intensive farming environments, where fish are often kept in high densities with shared water sources, infections can spread rapidly [24, 25]. Besides bacterial diseases, fungal, parasitic, and viral infections are also major threats, as they frequently weaken the fish’s immune system [26]. Therefore, quick and accurate detection of fish infections is essential for preventing large outbreaks and minimizing financial damage. Image analysis is one such method that has gained growing interest with the use of DL as an alternative to traditional diagnostic techniques. Building on previous research, a proposed CNN and pre-trained models were employed to assess the classification of infected freshwater fish species. The results showed that these models could identify and categorize four different fish species in a multi-class classification.

### Practical deployment considerations

For practical deployment considerations, additional augmentation techniques such as jitter and noise should be incorporated to account for domain variability across farms and imaging devices. In practice, this approach can increase confidence-threshold outputs combined with human-in-the-loop evaluation for low-confidence scenarios. The best-performing model can also be measured for on-device (mobile) inference using the same preprocessing steps applied during training. In future work, planned external validation across additional farms aims to further examine model robustness and sustainability.

### Performance comparison of DL models

In this study, a multi-class classification of four infected fish species was performed using a proposed CNN architecture with three convolutional layers, and it was compared to VGG16, InceptionV3, and ResNet50 with SGD as the optimizer. The performance evaluation showed that VGG16 (43.18%) and InceptionV3 (56.82%) achieved higher accuracy than the proposed CNN model (38.64%) after training for 20 epochs, with no evidence of overfitting or underfitting (Figures 4A–C, Table 1). The confusion matrix analysis of VGG16 and InceptionV3 revealed that they correctly predicted a total of 19 images, while the proposed CNN correctly predicted 17 images (Figures 5A–C). Conversely, InceptionV3 showed the highest misclassification rate, with 3–8 images misclassified (Figure 5C), followed by the poor performance of the proposed CNN model, in which SHF images were not classified correctly (Figure 5A). Overall, the results indicate that two models, VGG16 and InceptionV3, among the four evaluated architectures, were well-trained on both the training and testing datasets and effectively capable of classifying images.

### Comparison with previous studies

A dataset of 90 images featuring both healthy and infected fish (red spot and white spot) was previously used to classify fish diseases with a CNN model. Healthy fish achieved an accuracy of 97.2%, followed by red spot (94.44%) and white spot (91.67%). The red spot and healthy fish categories showed a sensitivity of 91.67%, while white spot showed a sensitivity of 83.33% due to false-negative rates. Specificity values were 95.83% for white spot and red spot and 91.67% for healthy fish. Overall, the mean accuracy, sensitivity, and specificity were 91.67%, 94.44%, and 97.2%, respectively, demonstrating that CNN models effectively identify and categorize fish diseases [14].

In another study, eight-class classification was conducted using 8000 augmented indigenous fish images with pre-trained models including VGG16, InceptionV3, MobileNet, and FishNet (a five-layer CNN model that used the Adam optimizer with ReLU and SoftMax activation functions). FishNet achieved an accuracy of 99%, followed by InceptionV3 at 100% and MobileNet at 99.83%, outperforming VGG16 which had 97.25% [27].

Similarly, another study used 1382 images categorized into four fish classes (white spot, black spot, red spot, and healthy fish) to evaluate nine classification algorithms, including DL models (CNN, VGG16, VGG19, and ResNet50), machine learning models, and ensemble methods that incorporate segmentation techniques to identify affected areas. The results showed that ResNet50 achieved an accuracy of 99.28%, while the ensemble model combining VGG16 and VGG19 achieved the highest accuracy of 99.64% [28].

Another study used ensemble methods combining artificial neural networks (ANNs), symbolic regression (SR), and decision trees (DTs) to predict fish mortality caused by four infectious diseases, based on real-world data from two large Greek fish farms. The results showed that hybrid models significantly outperformed individual ANN and SR models. Specifically, the final ensemble models achieved mean absolute error values of 52.18, 13.24, 2.95, and 1.27 for *Pasteurella*, *Vibrio harveyi*, *Myxobacteria*, and viral nerve necrosis, respectively [29].

Another study used the Random Forest machine learning algorithm to predict real-time fish mortality risk across five levels, from low to high. This model combined real-time sensor data with daily fish mortality reports from farmers and water quality parameters such as seawater temperature, salinity, conductivity, chlorophyll-a, turbidity, and dissolved oxygen. The model achieved an overall accuracy of 78.6% and precision values of 70% or higher for each risk level. Seawater temperature, salinity, and turbidity were identified as key predictors of fish mortality risk. These systems can help farm managers make daily decisions about labor and feeding management [30].

### Interpretation of current study findings

Using a balanced dataset with 80%–20% dataset splitting and on-the-fly augmentation, the CNN models evaluated in this study achieved an accuracy range of 38.64%–56.82%. These findings show that VGG16 and InceptionV3 outperformed the proposed CNN model. These results align with previous studies (Table 3) [14, 27, 28] that used imbalanced or large datasets and reported accuracy values from 91.67% to 100%. In contrast, this study used only 54 images per class.

**Table 3:** Comparison of model performance between the present study and previous studies using binary and multi-class fish image datasets.

Dataset description	Sample source	Model	Accuracy (%)	Reference
Healthy and two types of fish diseases (red spot and white spot)	Malaysia	CNN	91.67	[14]
Eight classes of indigenous fish	Bangladesh	FishNet	99	[27]
		InceptionV3	100	
		MobileNet	99.83	
		VGG16	97.25	
White spot, black spot, red spot, and healthy fish	Public datasets (Kaggle)	VGG16 + VGG19 ensemble	99.64	[28]
		ResNet50	99.28	
Four classes of infected fish: Asian seabass, red tilapia, snakeskin gourami, and snakeheads	Farms in Thailand	CNN	38.64	This study
		VGG16	43.18	
		InceptionV3	56.82	
		ResNet50	25	

In a previous study by our research group, a balanced dataset comprising six multi-class categories of protozoan parasites (790 images per class) with an 80%–20% split achieved an accuracy of 94% using a three-layer CNN with SGD as the optimizer [21]. These findings suggest that dataset size and image quality may be critical factors influencing model accuracy.

The superior performance of InceptionV3 compared with other models may be attributed to dataset-specific or context-dependent factors, including pattern complexity, class balance, image resolution, and dataset size. Additionally, the architecture of InceptionV3 enables parallel convolution operations with multiple kernel sizes (1 × 1, 3 × 3, and 5 × 5) alongside max-pooling layers. This design enables the extraction of diverse feature representations and may improve robustness when identifying key image features despite complex backgrounds, occlusions, and variations in lighting conditions and viewing angles. In contrast, ResNet50 may be more sensitive to regional redundancy during feature extraction.

Furthermore, using artificial intelligence techniques in aquaculture and fisheries management can be enhanced by ensemble methods and machine learning models that show high predictive accuracy [29, 30].

### Limitations and One Health implications

The severity of lesions was not evaluated in this study because it varied across different anatomical locations. This limitation is recognized and suggests an area for future methodological improvements. The study also emphasizes the importance of the One Health concept. Early visual assessment of diseased fish can reduce diagnosis time, help with laboratory confirmation, and lower unnecessary antimicrobial use. These improvements can boost aquatic animal health and productivity while supporting food security and public health through better pathogen monitoring in aquaculture systems.

The dataset used in this study represents a single-country setting (six provinces in Thailand) and includes four freshwater fish species exhibiting bacterial-type lesions. Viral and parasitic infections were rare and were not consistently confirmed during the study period. Although an input size of 200 × 200 × 3 was used with preprocessing and augmentation procedures, some degree of domain shift may still occur when images are captured using different cameras or under varying environmental conditions. Additionally, the results of this study are descriptive in nature, based on a fixed 80%–20% dataset split, and paired statistical significance tests were not conducted.

## CONCLUSION

This study assessed the performance of a lightweight three-layer CNN and three pre-trained DL architectures (VGG16, InceptionV3, and ResNet50) for multi-class classification of infected freshwater fish species using a balanced dataset from farm-level observations in Thailand. The results showed that InceptionV3 achieved the highest classification accuracy (56.82%), followed by VGG16 (43.18%) and the proposed lightweight CNN model (38.64%), while ResNet50 performed comparatively worse (25%). Confusion matrix analysis further confirmed that InceptionV3 correctly classified the most images among all evaluated models, highlighting its stronger feature extraction ability for identifying lesion patterns across different fish species. These findings suggest that pre-trained CNN architectures can improve classification performance when working with limited image datasets.

From a practical standpoint, incorporating deep learning–based image analysis into aquaculture health monitoring systems offers a promising way to quickly visually screen for diseased fish. These tools could help farmers and aquatic health professionals detect early signs of disease, allowing for timely interventions and prioritizing laboratory diagnostic tests. In resources-limited aquaculture settings where access to advanced lab diagnostics may be restricted, lightweight CNN models could serve as an effective initial screening method that can be run on mobile or edge devices for decision-making in the field.

A major strength of this study is the use of real-world images collected from commercial aquaculture farms across multiple provinces in Thailand, reflecting practical farm conditions rather than highly controlled laboratory datasets. The development of a balanced multi-class dataset, combined with on-the-fly data augmentation, also enabled evaluation of model performance under realistic data limitations commonly encountered in aquaculture disease monitoring. In addition, the comparative analysis of lightweight and pre-trained architectures provides useful insights for selecting suitable models for aquaculture health surveillance systems.

However, the relatively small dataset size and limited species coverage are significant constraints that may affect classification accuracy. Future research should aim to expand the dataset to include a broader variety of fish species, disease conditions, and environmental settings. Including viral and parasitic disease cases, using ensemble or hybrid machine learning methods, and performing external validation across multiple farms and geographic regions would further strengthen the robustness and generalizability of the models. Incorporating domain adaptation techniques and uncertainty estimation could also improve model reliability in real-world applications.

In conclusion, this study highlights the potential use of DL models, especially InceptionV3 and VGG16, for automatically classifying infected freshwater fish species through image analysis. While further refinement and validation are necessary, these models offer a promising step toward creating smart, affordable disease monitoring systems that can aid sustainable aquaculture, enhance fish health surveillance, and support broader One Health initiatives.

## DATA AVAILABILITY

Raw images are not accessible to the public due to farm confidentiality.

## AUTHORS’ CONTRIBUTIONS

ES: Data collection, conceptualization, experiment design, manuscript drafting, and revision. SN: Conceptua-lization, data curation, methodology, investigation, data analysis, visualization, validation, manuscript drafting, and revision. VS: Conceptualization, manuscript drafting, revision, and project administration. CR: Conceptua-lization, supervision, project administration, funding acquisition, manuscript drafting, and revision. All authors have read and approved the final manuscript.

## References

[1] Dongyu Q (2024). The state of world fisheries and aquaculture - blue transformation in action. Rome: Food and Agriculture Organization of the United Nations.

[2] Food and Agriculture Organization of the United Nations (2024). Global fisheries and aquaculture production reaches a new record high [Internet].

[3] Ohia CMD (2025). Aquaculture technologies and practices: balancing innovation, environment and economy for sustainability. In: Food security, nutrition and sustainability through aquaculture technologies.

[4] Food and Agriculture Organization of the United Nations (2022). The state of world fisheries and aquaculture 2022.

[5] Amillano-Cisneros JM, Fuentes-Valencia MA, Leyva-Morales JB, Savín-Amador M, Márquez-Pacheco H, de Jesús Bastidas-Bastidas P (2025). Effects of microorganisms in fish aquaculture from a sustainable approach: a review. Microorg.

[6] Austin B, Austin DA (2016). Bacterial fish pathogens: disease of farmed and wild fish.

[7] Bondad-Reantaso MG, MacKinnon B, Karunasagar I, Fridman S, Alday-Sanz V, Brun E (2023). Review of alternatives to antibiotic use in aquaculture. Rev Aquac.

[8] Subasinghe RP, Delamare-Deboutteville J, Mohan CV, Phillips MJ (2019). Vulnerabilities in aquatic animal production. Rev Sci Tech.

[9] Gangil P, Paul MK, Mondal D, Kumari S, Prasad PR, KE V (2025). Melioidosis molecular diagnostics: an update. Virulence.

[10] Mabrok M, Elayaraja S, Chokmangmeepisarn P, Jaroenram W, Arunrut N, Kiatpathomchai W (2021). Rapid visualization in the specific detection of *Flavobacterium columnare*, a causative agent of freshwater columnaris using a novel recombinase polymerase amplification (RPA) combined with lateral flow dipstick (LFD) assay. Aquac.

[11] Adams A, Thompson KD (2011). Development of diagnostics for aquaculture: challenges and opportunities. Aquac Res.

[12] Martínez-Porchas M, Vargas-Albores F (2017). Microbial metagenomics in aquaculture: a potential tool for a deeper insight into the activity. Rev Aquac.

[13] Wu J (2017). Introduction to convolutional neural networks.

[14] Hasan N, Ibrahim S, Azlan AA (2022). Fish diseases detection using convolutional neural network (CNN). Int J Nonlinear Anal Appl.

[15] Kaya V, Akgül İ, Tanır ÖZ (2023). IsVoNet8: a proposed deep learning model for classification of some fish species. J Agric Sci.

[16] Gupta A, Bringsdal E, Knausgård KM, Goodwin M (2022). Accurate wound and lice detection in Atlantic salmon fish using a convolutional neural network. Fishes.

[17] Mia MJ, Mahmud RB, Sadad MS, Al Asad H, Hossain R (2022). An in-depth automated approach for fish disease recognition. J King Saud Univ - Comput Inf Sci.

[18] Sun Y, Wong AK, Kamel MS (2009). Classification of imbalanced data: a review. Int J Pattern Recognit Artif Intell.

[19] Cruz RM, Souza MA, Sabourin R, Cavalcanti GD (2019). Dynamic ensemble selection and data preprocessing for multi-class imbalance learning. Int J Pattern Recognit Artif Intell.

[20] Potharaju SP, Sreedevi M, Ande VK, Tirandasu RK (2019). Data mining approach for accelerating the classification accuracy of cardiotocography. Clin Epidemiol Glob Health.

[21] Elayaraja S, Yeruva S, Stejskal V, Nandipati S (2024). Multi-class microscopic image analysis of protozoan parasites using convolutional neural network. J Univers Comput Sci.

[22] Bisong E (2019). Building machine learning and deep learning models on the Google cloud platform.

[23] 365 Data Science. Cross-entropy loss [Internet] [place unknown]: 365 Data Science.

[24] Elayaraja S, Liu G, Zagorsek K, Mabrok M, Ji M, Ye Z (2021). TEMPO-oxidized biodegradable bacterial cellulose (BBC) membrane coated with biologically-synthesized silver nanoparticles (AgNPs) as a potential antimicrobial agent in aquaculture (in vitro). Aquac.

[25] Syanya FJ, Mahadevan H, Khanna AN, Mathia WM, Mumina P, Litabas JA (2025). Biosecurity protocols and fish health management in Kenyan fish hatcheries:a key to sustainable production of quality fish seed. Mar Fish Sci.

[26] Rodrigues T, Guardiola FA, Almeida D, Antunes A (2025). Aquatic invertebrate antimicrobial peptides in the fight against aquaculture pathogens. Microorg.

[27] Dey K, Hassan MM, Rana MM, Hena MH (2021). Bangladeshi indigenous fish classification using convolutional neural networks. In: 2021 Int Conf Inf Tech (ICIT), Amman, Jordan.

[28] Mamun MR, Rahman US, Akter T, Azim MA (2023). Fish disease detection using deep learning and machine learning. Int J Comput Appl.

[29] Aravanis T, Hatzilygeroudi I, Spiliopoulos G (2024). Ensemble modelling for predicting fish mortality. Appl Sci.

[30] Saville R, Fujiwara A, Hatanaka K, Wada M, Yaman A, Puspasari R (2026). AI-powered decision support system for mariculture: real-time fish mortality prediction with random forest. Aquac Eng.

